# The Imprinted Gene DIO3 Is a Candidate Gene for Litter Size in Pigs

**DOI:** 10.1371/journal.pone.0031825

**Published:** 2012-02-29

**Authors:** Albart Coster, Ole Madsen, Henri C. M. Heuven, Bert Dibbits, Martien A. M. Groenen, Johan A. M. van Arendonk, Henk Bovenhuis

**Affiliations:** Animal Breeding and Genomics Group, Wageningen University, Wageningen, The Netherlands; Aarhus University, Denmark

## Abstract

Genomic imprinting is an important epigenetic phenomenon, which on the phenotypic level can be detected by the difference between the two heterozygote classes of a gene. Imprinted genes are important in both the development of the placenta and the embryo, and we hypothesized that imprinted genes might be involved in female fertility traits. We therefore performed an association study for imprinted genes related to female fertility traits in two commercial pig populations. For this purpose, 309 SNPs in fifteen evolutionary conserved imprinted regions were genotyped on 689 and 1050 pigs from the two pig populations. A single SNP association study was used to detect additive, dominant and imprinting effects related to four reproduction traits; total number of piglets born, the number of piglets born alive, the total weight of the piglets born and the total weight of the piglets born alive. Several SNPs showed significant (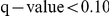) additive and dominant effects and one SNP showed a significant imprinting effect. The SNP with a significant imprinting effect is closely linked to DIO3, a gene involved in thyroid metabolism. The imprinting effect of this SNP explained approximately 1.6% of the phenotypic variance, which corresponded to approximately 15.5% of the additive genetic variance. In the other population, the imprinting effect of this QTL was not significant (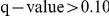), but had a similar effect as in the first population. The results of this study indicate a possible association between the imprinted gene DIO3 and female fertility traits in pigs.

## Introduction

Genomic imprinting is an epigenetic phenomenon where the degree of expression of an allele depends on its parental origin. The parent-of-origin-dependent allele expression of genomically imprinted genes is controlled by epigenetic marks such as DNA methylation and histone modifications which are established during gametogenesis and mostly maintained during life [1], [2].

Genomic imprinting has been found in viviparous mammals and in seeded plants [3], [4]. To date, more than 100 imprinted genes have been experimentally identified in mammals (http://igc.otago.ac.nz and http://www.geneimprint.com/site/genes-by-species), several hundreds of genes have been predicted to be imprinted in human and mouse [5], [6] and recently as many as 1300 loci with parent-of-origin-dependent allele expression have been identified in the mouse brain [7], [8].

The majority of genomically imprinted genes are found in clusters containing protein coding and non-coding genes [9], [10]. Imprinted genes play important roles in development of the placenta, in fetal growth and development and in neurological development. Hence, aberrant allele-specific expression of imprinted genes can disrupt prenatal development and is associated with different genetic diseases including several forms of cancer and a number of neurological disorders [9], [11]. Some imprinted genes are imprinted in all tissues throughout all stages of development whereas others are imprinted in a tissue or sex specific manner, at a particular stage of development or display opposite imprinting in different tissues [7], [8], [12]–[14]. Comparative studies indicate a marked difference in genomic imprinting among singleton and polytocous species, particularly for genes imprinted in the placenta [15], [16] and high expression of the majority of imprinted genes tested to date has been demonstrated in extraembryonic tissues, suggesting a critical role for imprinted genes in placental development [17].

At the phenotypic level, imprinting is manifested through a contrast between the two heterozygote classes that exist for a genotype (AB and BA classes, in this notation the first letter of the genotype indicates the allele inherited from the mother and the second letter the allele inherited from the father) [18], which both contribute to the total phenotypic variation of a trait. This variation has been exploited in QTL (Quantitative Trait Loci) mapping studies, which associate marker genotype classes to phenotypic variation. Adapting QTL-linkage mapping to imprinting in livestock animals was first described by Knott et al. [19], and shortly thereafter applied in a genome-wide scan for imprinted QTL by de Koning et al. [20]. This stimulated a variety of imprinting QTL studies in livestock animals, especially in pigs where 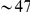 imprinted QTL, related to a broad scale of phenotypic traits, have been described [20]–[26]. The reported imprinted QTL are scattered over all of the pig chromosomes except one, and cover a variety of traits such as meat quality and reproduction (see http://igc.otago.ac.nz for an overview).

A common denominator in genome screens for imprinted QTL in pigs is the use of experimental crosses between divergent pig breeds or lines. When the lines are not completely inbred, this incurs the risk of false positive detection of imprinted QTL due to heterogeneity in the original purebred populations [27]. Further, this approach might detect QTL that are fixated within commercial lines and hence have no value for selective breeding within those commercial lines.

One of the most intensively studied imprinted QTL in pigs is the paternally expressed QTL on chromosome 2, which affects heart muscle size, muscle growth and fat deposition [20], [28], [29]. This imprinted QTL maps to a region that includes the imprinted IGF2 gene. Sequencing of the IGF2 gene in different pig breeds and wild boars showed that the QTL is caused by a G to A nucleotide change in a CpG island in intron 3 of this gene [30]. This substitution increases the expression of IGF2 in postnatal muscle and is responsible for the observed phenotypic effect.

Several hypotheses for the evolution of genomic imprinting have been formulated, many related to allocation of resources from mother to offspring during the early stages of development. These hypotheses include: the parental conflict hypothesis that explains genomic imprinting by a parental conflict in allocation of resources to the offspring [31]; the intralocus sexual conflict hypothesis based on the idea that natural selection should favor paternal expression in males and maternal expression in females [32] and the co-adaptation theory explaining genomic imprinting as a result of the evolution of coadaptation between mother and offspring traits [33].

The presumption that genomically imprinted genes regulate the resource allocation between mother and offspring [31]–[33], together with the important role of genomic imprinting in placental and embryonic development suggests a possible involvement of imprinted genes in mammalian female fertility traits. Identification of genomically imprinted QTL involved in these traits would therefore add to the knowledge of genomic imprinting and would also disclose possibilities for animal breeding, especially if these traits could be manageable in a sex specific manner.

The aim of this study was therefore to explore whether putative imprinted genes or regions associate with fertility traits in commercial pigs. For this purpose, fifteen evolutionary conserved imprinted regions were genotyped in two commercial pig breeds. An association study was used to detect additive, dominant and imprinting effects related to four reproduction traits (total number of piglets born (TB), the number of piglets born alive (LB), the total weight of the piglets born (TW) and the total weight of the piglets born alive (LW)). Several additive and dominant associations and one imprinted association were detected. These results are discussed in relation to their biological relevance.

## Results

### Description of data

The data of two commercial purebred pig populations were analyzed in this study. Both populations were Large White dam lines which have been selected for several generations for commercially important traits, including reproduction traits. The traits analyzed in this study were reproductive performance of the sows, based on their litters. Some of the litters were purebred and others were crossbreds. Phenotypes considered were the total number of piglets born (TB), the number of piglets born alive (LB), the total weight of the piglets born (TW) and the total weight of the piglets born alive (LW). Table 1 summarizes the characteristics of the two pig populations. In population C1, 736 individuals were genotyped, of which 490 had phenotypes for at least one trait (Table 1). In population C2, 1078 individuals were genotyped, of which 983 had phenotypes for at least one of the traits (Table 1). The number of genotyped sows with observations for LW and TW was especially low in population C1 (Table 1).

**Table 1:** Descriptive statistics for the populations.

Population	Trait	N. phenotypes.	N. genotypes.	Mean parity n.	Mean	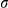
**C1**						
	LB	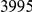	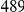	2.35	13.07	2.85
	LW	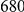	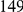	2.57	18.36	4.07
	TB	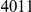	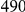	2.35	14.05	2.91
	TW	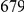	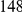	2.57	19.86	4.06
**C2**						
	LB	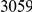	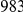	2.47	13.59	2.94
	LW	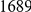	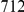	2.81	17.39	3.70
	TB	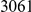	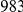	2.47	14.74	3.07
	TW	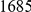	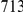	2.82	18.90	3.75

N. phenotypes = number of sows with phenotyp ic data; N. genotypes = number of sows with genotyp ic and phenotyp ic data; Mean parity n = mean parity number corresponding to the phenotypes in the data; Mean = mean of the phenotype data, averaged over all parities; 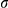 = (uncorrected) standard deviation of the phenotype data. The traits included in the analyses were: LB = number of piglets born alive in a litter, LW = weight of the liveborn piglets in a litter in kg; TB = number of piglets born in a litter; TW = weight of the piglets born in a litter in kg.

Table 2 shows the variance components and the heritability estimate s for the four traits in populations C1 and C2. In general, the additive genetic component (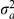) contributed more to the phenotypic variation than the permanent environmental (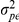) or maternal (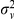) effects. The variance due to maternal effects was low for all traits. The heritability estimates for the traits were moderate to low. The heritability estimates for LW and TB differed between the population, however the confidence intervals for the heritability estimates overlap (Table 2)

**Table 2:** Variance components estimated.

Population	Trait	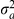	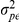	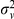	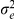	h 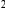
**C1**						
	LB	0.78 (0.14)	0.73 (0.13)	0.06 (0.06)	6.51 (0.11)	0.10 (0.02)
	LW	3.13 (0.80)	0.87 (0.65)	0.18 (0.35)	10.03 (0.51)	0.22 (0.05)
	TB	0.76 (0.14)	0.62 (0.12)	0.11 (0.06)	6.41 (0.11)	0.10 (0.02)
	TW	3.51 (0.78)	0.68 (0.60)	0.09 (0.30)	8.70 (0.45)	0.27 (0.05)
**C2**						
	LB	1.02 (0.21)	0.48 (0.14)	0.08 (0.06)	6.73 (0.11)	0.12 (0.02)
	LW	1.70 (0.55)	1.73 (0.38)	0.12 (0.18)	8.88 (0.25)	0.14 (0.04)
	TB	1.48 (0.26)	0.60 (0.16)	0.09 (0.07)	6.90 (0.12)	0.16 (0.03)
	TW	3.03 (0.58)	1.44 (0.41)	0.00 (0.00)	7.81 (0.22)	0.25 (0.04)

Additive variance (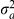), permanent environment variance (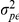), variance of the maternal effects (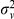), residual variance (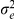) and heritability (h
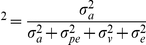
) (with standard errors) estimated for the four traits in populations C1 and C2. The traits included in the analyses were: LB = number of piglets born alive in a litter, LW = weight of the liveborn piglets in a litter in kg; TB = number of piglets born in a litter; TW = weight of the piglets born in a litter in kg.

### Characteristics of the SNPs

The fifteen selected regions are located on ten different chromosomes with three regions on chromosome 1, two regions on chromosomes 2, 9, and 17 and one region on chromosomes 5, 6, 7, 8, 14 and 18 (Table 3). The size of the regions varied between 0.55 and 4 Mb and the smallest distance between two regions on one chromosome was approximately 14.5 MB, making any linkage disequilibrium (LD) between two regions unlikely. Between 20 to 38 SNPs were genotyped in the different regions (see the Material and Methods section for details). After excluding monomorphic SNPs and SNPs with parental errors and SNPs that failed during genotyping, the number of polymorphic markers varied between 13 in region 9_2 to 32 in region 9_1 (Table 3) with generally the same markers being polymorphic in both populations. The minor allele frequency (MAF) of the SNPs was usually higher in population C1 than in C2 and the average LD between adjacent SNPs was lower in population C1 than in C2 (Table 3). This indicates that population C2 was genetically less variable in the genotyped regions than population C1.

**Table 3:** Summary of the regions.

Region			Population C1			Population C2		
	Begin	Size	nsnp	MAF	r 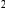	nsnp	MAF	r 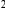
1_1	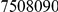	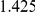	26	(0.16 0.28 0.40)	(0.02 0.10 0.13)	26	(0.15 0.25 0.35)	(0.02 0.24 0.33)
1_2	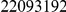	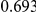	14	(0.10 0.26 0.37)	(0.01 0.09 0.20)	14	(0.05 0.15 0.24)	(0.01 0.14 0.15)
1_3	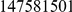	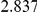	28	(0.08 0.22 0.39)	(0.04 0.27 0.57)	25	(0.03 0.16 0.31)	(0.01 0.30 0.68)
2_1	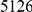	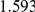	30	(0.18 0.24 0.34)	(0.04 0.31 0.48)	31	(0.01 0.10 0.19)	(0.01 0.24 0.29)
2_2	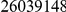	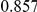	18	(0.24 0.31 0.38)	(0.02 0.26 0.48)	18	(0.16 0.26 0.38)	(0.04 0.39 0.65)
5_1	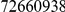	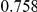	15	(0.12 0.27 0.42)	(0.00 0.18 0.17)	15	(0.10 0.20 0.31)	(0.00 0.17 0.24)
6_1	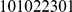	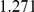	14	(0.18 0.26 0.40)	(0.00 0.17 0.19)	14	(0.31 0.32 0.47)	(0.01 0.16 0.22)
7_1	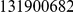	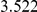	28	(0.22 0.30 0.42)	(0.04 0.20 0.30)	28	(0.15 0.24 0.32)	(0.01 0.26 0.45)
8_1	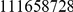	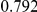	16	(0.19 0.27 0.33)	(0.01 0.18 0.29)	16	(0.11 0.21 0.27)	(0.01 0.20 0.35)
9_1	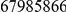	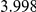	32	(0.17 0.28 0.38)	(0.06 0.33 0.66)	32	(0.12 0.17 0.20)	(0.11 0.44 0.76)
9_2	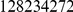	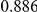	13	(0.15 0.25 0.34)	(0.03 0.15 0.16)	13	(0.05 0.14 0.21)	(0.00 0.19 0.26)
14_1	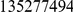	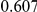	16	(0.31 0.34 0.38)	(0.17 0.39 0.55)	16	(0.40 0.38 0.41)	(0.34 0.53 0.79)
17_1	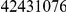	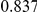	17	(0.11 0.22 0.34)	(0.08 0.36 0.57)	17	(0.19 0.20 0.21)	(0.68 0.74 0.94)
17_2	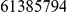	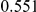	19	(0.20 0.30 0.45)	(0.02 0.25 0.40)	19	(0.21 0.27 0.39)	(0.01 0.26 0.38)
18_1	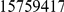	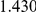	19	(0.30 0.35 0.48)	(0.01 0.30 0.53)	19	(0.12 0.18 0.23)	(0.15 0.44 0.71)

The regions are named as chromosome_region (regions numbered from 1 to n at each chromosome). Begin position of the region in bp (Begin); size of the region in Mb (Size); number of polymorphic SNP markers in each population (nsnp); first quartile, mean, and third quartile of the minor allele frequency in each population (MAF); first quartile, mean, and third quartile of the linkage disequilibrium between adjacent polymorphic markers in each population measured as 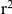. Position and size of the region were calculated from build 9 of the pig genome.

### Marker effects

**Figure 1:**
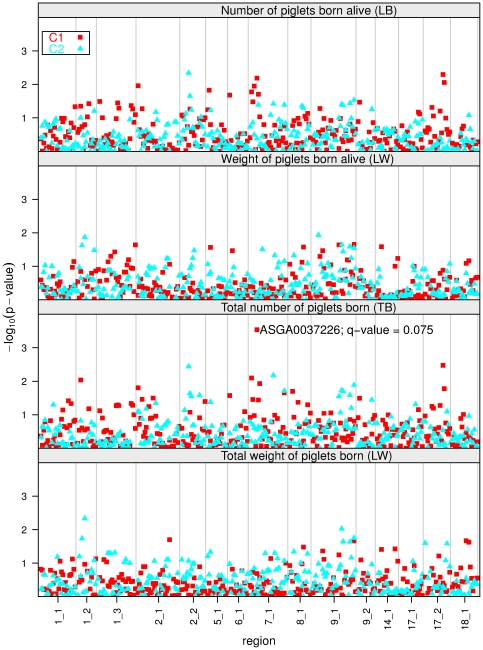
Plot of the 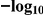
(p-value) of imprinting effects for the four traits in populations C1 and C2. The vertical lines separate the regions. The marker with a 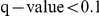 in region 7_1 for trait TB is indicated. See the supplemental file S1 for the corresponding p-values of individual markers.

Single SNP association analyses were performed to detect additive, dominance and imprinting effects related to the four traits. For each combination of trait and population, several additive, dominant and imprinted effects had a 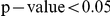 (see supplemental file S1). The p-values for the imprinting effects of the markers are shown in Figure 1.

Table 4 shows the number of markers in a region with a 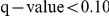 for each trait in each population. Significant effects were found in eight of the fifteen regions. There were considerable differences in number and type of effects between the two populations (Table 4). In population C1, three dominance and one imprinting effect were found while in population C2 several additive effects and two dominance effects were found (Table 4). The absence of effects with a 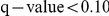 for traits LW and TW in population C1 is probably a result of the small number of observations for these traits in this population. Of the regions with a significant effect region 7_1 seems most interesting because it contained a significant imprinted effect for trait TB in population C1 and for population C2 it contained several significant additive effects for the four traits (Table 4).

**Table 4:** Significant associations from the single marker analyses.

Population		Region							
	Trait	1_1	1_3	2_2	7_1	8_1	14_1	17_2	18_1
**C1**									
	LB		1D						
	LW								
	TB		1D		1I		1D		
	TW								
**C2**									
	LB			1D	2A				
	LW				7A				1A
	TB	1A	8A	1D	2A				1A
	TW	3A	8A		8A	1A	1A	1A	1A

Number of markers with 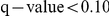 for the additive (A), dominance (D), or imprinting (I) in each region and for each population. The traits included in the analyses were: LB = number of piglets born alive in a litter, LW = weight of the liveborn piglets in a litter in kg; TB = number of piglets born in a litter; TW = weight of the piglets born in a litter in kg. See Table 3 for explanation of the regions. See the supplemental file S1 for the corresponding p-values of individual markers.

The imprinting effect in population C1 with significant FDR in region 7_1 on trait TB corresponded to SNP marker ASGA0037226. In this population, this region contained several other markers with small 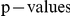 for imprinting effects on traits TB and LB, but none of these effects had a 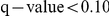.

The significant imprinting effect in region 7_1 on trait TB in population C1 explained 1.6% of the phenotypic variance of trait TB (Table 5), which represents approximately 15.5% of the additive genetic variance of this trait (with h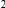 of 0.1, Table 2). This marker explained a large percentage of the phenotypic variance of the trait when it was compared to the percentage of the phenotypic variance explained by the imprinting effects of other markers (Table 5). The most significant additive effects in this region in population C2 explained 0.9% and 2.3% of the phenotypic variance, corresponding to 3.8% and 16.1% of the additive genetic variance of these traits (Table 5).

**Table 5:** Phenotypic variance (in %) explained by the most significant marker in each region for the additive, dominance and imprinting effect.

Term		C1				C2			
	Region	LB	LW	TB	TW	LB	LW	TB	TW
**A**									
	1_1	0.36	2.81	0.60	3.70	0.49	0.64	**0.48**	**1.00**
	1_3	0.61	2.40	0.90	5.65	0.46	0.77	**0.52**	**1.76**
	2_1 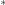	0.20	39.48	0.26	11.84	0.16	0.19	0.31	0.40
	2_2	1.73	2.84	0.19	3.16	0.01	0.00	0.41	0.00
	7_1	1.25	1.21	0.61	0.12	**1.20**	**2.26**	**1.20**	**0.94**
	8_1	2.22	8.06	0.87	5.98	0.73	0.64	0.70	**1.55**
	14_1	0.47	3.04	0.39	3.87	0.11	0.54	0.30	**0.75**
	17_2	1.31	2.64	0.30	0.67	0.32	0.12	0.31	**0.24**
	18_1	0.49	0.06	0.76	0.22	0.00	**0.56**	**0.16**	**0.18**
**D**									
	1_1	0.47	2.52	0.51	1.98	4.64	0.91	4.45	0.55
	1_3	**3.48**	2.99	**2.97**	1.75	0.07	0.41	0.11	0.65
	2_1 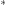	0.30	24.91	0.56	1.84	1.65	0.25	1.69	0.22
	2_2	0.73	1.15	0.37	2.67	**0.58**	0.46	**0.67**	0.33
	7_1	0.44	3.65	1.46	3.34	0.18	0.30	0.23	1.01
	8_1	0.76	1.42	1.07	1.73	0.31	1.10	0.13	0.30
	14_1	1.08	4.84	**1.45**	4.40	0.56	1.48	0.43	1.14
	17_2	1.38	2.38	1.23	0.42	0.10	0.61	0.19	0.49
	18_1	2.95	0.45	0.33	0.85	0.39	1.04	0.63	2.76
**I**									
	1_1	0.37	3.13	0.42	2.02	0.12	0.57	0.21	0.39
	1_3	0.57	2.26	0.45	1.87	0.24	0.76	0.05	0.78
	2_1 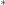	0.88	1.20	0.73	2.85	0.16	0.41	0.21	0.34
	2_2	0.42	1.49	0.36	1.45	0.37	0.42	0.42	0.33
	7_1	0.92	0.91	**1.55**	1.44	0.24	0.68	0.45	0.41
	8_1	0.45	2.40	0.77	2.20	0.25	0.41	0.11	0.53
	14_1	0.17	2.76	0.31	12.00	0.08	0.72	0.13	0.19
	17_2	0.95	1.18	0.96	5.07	0.03	0.45	0.15	0.52
	18_1	0.30	1.38	0.43	2.83	0.18	0.21	0.16	0.18

Variance of the additive (A), dominance (D) and imprinting effect (I) of the most significant marker in each region, expressed as percentage of the total phenotypic variance. The bold figures indicate the effects with a 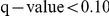. The traits included in the analyses were: LB = number of piglets born alive in a litter, LW = weight of the liveborn piglets in a litter in kg; TB = number of piglets born in a litter; TW = weight of the piglets born in a litter in kg. 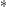 region 2_1 was included in the t able because it contains the imprinted IGF2 gene, for which an effect on sow prolificacy was found (see Discussion). See Table 3 for and explanation of the regions.

**Figure 2:**
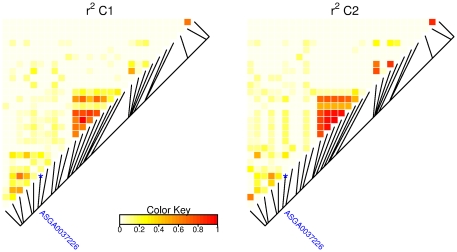
Linkage disequilibrium in region 7_1, calculated as 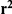. The highlighted SNP marker ASGA0037226 was the marker with the significant imprinting effect in population C1.

Estimates for LD in region 7_1 (Figure 2) revealed weak LD between marker ASGA0037226 and other markers in this region, explaining why the markers neighboring marker ASGA0037226 did not reach significance on trait TB in population HG. Noteworthy is the strong LD of six to seven SNP markers in another part of region 7_1 (Figure 2), which was especially apparent in population C2 but could also be observed in population C1. This block of SNPs corresponded to the SNPs with significant additive effects in population C2 (Table 4).

### Imprinted marker in region 7_1

Table 6 summarizes the unadjusted means for the ASGA0037226 genotype classes and the additive, dominance and imprinting effects estimated using Equation 1. The estimated imprinting effects were positive for litter size in both populations, thus consistently pointing to the same mode of imprinting (although only the effect on trait TB in population C1 was significant). In population C1, the positive imprinting effects for the four traits agreed with the unadjusted means of the two genotype classes; heterozygote individuals with a maternal B allele had larger and heavier litters than heterozygote individuals with a paternal B allele. Thus, the imprinting pattern for the trait TB suggests maternal expression with the maternal B allele resulting in larger litter size than the maternal A allele. Notably, the frequency of the BA genotype was higher in both populations than that of the AB genotype and genotype frequencies deviated from the expected frequencies under Hardy Weinberg Equilibrium.

**Table 6:** Unadjusted population means and regression coefficients for genotypes of marker ASGA0037226 in region 7_1.

Pop.		Genotype class				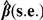		
	Trait	AA	BA	AB	BB	A	D	I
**C1**								
	LB	12.06 (18)	12.69 (106)	11.85 (60)	12.17 (314)	−0.06 (0.23)	0.22 (0.27)	0.44 (0.16)
	LW	15.48 (5)	17.33 (21)	17.29 (10)	14.69 (44)	0.01 (0.51)	1.34 (0.70)	0.15 (0.49)
	TB	12.72 (18)	13.63 (107)	12.34 (61)	13.10 (316)	−0.23 (0.23)	−0.17 (0.27)	0.58 (0.16)
	TW	16.23 (5)	19.10 (21)	18.05 (10)	15.87 (44)	−0.20 (0.50)	1.28 (0.68)	0.61 (0.48)
**C2**								
	LB	12.60 (5)	12.42 (91)	12.38 (63)	12.29 (838)	−0.51 (0.40)	−0.38 (0.41)	0.13 (0.16)
	LW	16.87 (4)	14.06 (29)	15.65 (22)	14.75 (233)	−0.23 (0.54)	−0.27 (0.57)	0.09 (0.25)
	TB	13.40 (5)	13.49 (91)	13.17 (63)	13.17 (840)	−0.35 (0.42)	−0.20 (0.44)	0.18 (0.17)
	TW	18.15 (4)	15.24 (28)	16.52 (22)	15.76 (234)	−0.40 (0.56)	−0.63 (0.59)	0.19 (0.26)

Summary of marker ASGA0037226 in region 7_1 which had a 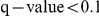 for the imprinting effect (Table 4 and Figure 1). Mean value of the first parity (number of observations) for each genotype class in the two populations. The first character of the genotype class is the allele of maternal origin, the second character is the allele of paternal origin. 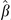
's are the estimated regression coefficients for the additive, dominance and imprinting effects. The traits included in the analyses were: LB = number of piglets born alive in a litter, LW = weight of the liveborn piglets in a litter in kg; TB = number of piglets born in a litter; TW = weight of the piglets born in a litter in kg.

To ensure that the observed imprinted effect was not an effect of a stochastic unequally assignment of parental alleles from heterozygotic parents, genotypic means were also calculated based on matings that resulted in irrefutable allele origin in the offspring (e.g a BA genotype from a AA mother and a BB father). In both populations, the means for LB and TB of the BA genotype where higher than those of the AB genotype, validating the imprinting effect (results not shown). The deviation from the expected Hardy-Weinberg equilibrium can be specific for the sampled populations and therefore we also estimated these deviations for the other markers. For this purpose, the 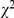 test statistic for ASGA0037226 was compared to the distribution of 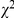
's test statistic of all markers. In population C1, 41% of the markers had a higher 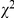 test statistic than ASGA0037226 and in population C2 this was 48%. This indicated that the genotype frequencies observed for marker ASGA0037226 were not significantly different from genotype frequencies observed for other markers in the data.

## Discussion

Fertility is an economically important trait in the pig breeding industry for which considerable selection has been applied in the last decades. Many studies have been conducted to find QTL and genes related to reproduction traits in pigs (see [34] for a recent review), but imprinted effects were not taking into account in the majority of these studies.

The developing placenta, together with the uterine environment, play critical roles in prenatal growth and survival. The observation that many imprinted genes have high expression in extraembryonic tissues [17], and the marked difference in the number of placental imprinted genes among singleton and polytocous species [15], [16], and the distinct hypotheses for the evolution of genomic imprinting [31]–[33], suggest a role for imprinted genes in placental development and in the regulation of litter size. Thus, we hypothesized that imprinted genes may affect pig reproduction traits such as litter size and/or litter weight. To test this hypothesis, fifteen evolutionary conserved imprinted regions were genotyped in two commercial pig breeds, followed by an association study with the objective to detect imprinted QTL affecting sow fertility traits.

We used a model similar to that of Hager et al. [18] for the analysis of the data. The model included additive and dominance effects in a addition to imprinting effects, which effectively corrects the imprinting effects for these additive and dominance effects and thus reduces the risk of false positive imprinting effects. In addition, we could estimate effects of the three genetic effects and thus compare the size of their effects. The model included random terms accounting for maternal, permanent environmental and polygenic effects. The inclusion of the maternal effects was motivated by the study of Santure et al. [35] and of Hager et al. [36], who showed possible confounding between maternal effects and imprinting effects.

Knowledge of the parental origin of marker alleles is essential for detection of genomic imprinting [18], [20], [37]. In our data, the parental origin of alleles was estimated using the program cvmhaplo [38], which reconstructs marker haplotypes based on pedigree and marker information. The accuracy of haplotypes reconstructed with this program was expected to increase with the number of offspring. For this reason, paternal halfsib groups of sows and their ancestors were selected for genotyping. By inferring the parental origin of alleles, litter records of all available sows could be used in the analyses without being limited to using sows of homozygous fathers or mothers only. The sizes of both populations were aimed at 1.000 individuals based on an initial power study, which showed that the power to detect an imprinted QTL that explained 1% of the phenotypic variance was 0.65 (using a type I error of 0.05 and without accounting for multiple testing).

To avoid a large number of false positive effects due to the large number of tests performed, the false discovery rate (FDR) was calculated. A consequence was that we used a stringent significance thresholds for our tests, leading to reduced power to detect imprinting effect, but strengthening the confidence in the detected effects. The fact that we only found significant evidence for one imprinted effect is partially due to this reduced power, but does also illustrate the challenge of detecting imprinted effects in association studies.

The proportion of phenotypic variance explained by this imprinted effect was substantial, accounting for 1.6% of the phenotypic variance (which is equivalent to 15.5% of the additive genetic variance of this trait in this population). In population C2, the imprinting effect of this marker was not significant, but the estimated imprinting effect had the same sign as in population C1 (Table 6).

We performed additional analyses using haplotypes instead of single SNP and fitting additive, dominance and imprinting effects as random effects. Results from this analysis show that the variance explained by imprinting effects was approximately equal to the imprinting variance based on the single SNP analysis. These results suggest that the SNP ASGA0037226 is in weak LD with other SNPs in this region and that the association between the QTL and these other SNPs is weak. This is in line with the LD pattern in region 7_1 (Figure 2)

Region 7_1 corresponds to the DLK1-DIO3 imprinted domain which contains at least three maternal imprinted protein coding genes (DLK1, RTL1 and DIO3) and many paternal imprinted small and large ncRNA genes. The SNP marker with significant imprinted effect (ASGA0037226) is located approximately 25 kb from the DIO3 gene and about 500 kb from other known imprinted genes in this region. DIO3 codes for type 3 deiodinase (D3), a selenoprotein that plays an important role in thyroid hormone metabolism. Thyroid hormones influence a wide variety of biological processes in vertebrates. Their importance is most evident during prenatal and early neonatal development (for references see Hernandez, 2005 [39]). D3 enzymatic activity inactivates T4 (a prohormone) and T3 (the biologically active thyroid hormone) into metabolites which are biologically inactive [40]. D3 displays a marked developmental pattern of expression. In both humans and rodents D3 is expressed at very high levels in the uterine decidual tissue in early pregnancy and in the uterine wall and placenta(s) later in pregnancy (reviewed in [39]). Since maternal levels of thyroid hormones are much higher during pregnancy than those in the developing offspring, it is assumed that D3 in uterine and placental tissues have a role in maintaining embryonic and fetal levels of thyroid hormones at an optimum level for optimal development and survival. DIO3 is partially maternally imprinted in mouse tissues (1∶4 maternal∶paternal expression) [41]–[44] and was recently found to be paternally expressed in several embryonic tissues and in 2-month-old pigs [45], [46]. Disruption of the imprinting status or knocking-out of DIO3 in mice affects D3 enzyme activity and results in abnormal embryonic thyroid hormone levels, abnormal embryonic development, lifetime marked growth retardation and low fertility rate [41], [42], [47]. In addition, the number of DIO3 double knock-out (D3KO) offspring from heterozygous crosses did not follow Mendelian expectations indicating partial embryonic lethality of D3KO mice. Thus, based on the effects of this gene and on the strong and consistent indications of imprinting of SNP ASGA0037226, this SNP could be in strong LD with DIO3 and hereby suggesting that DIO3 plays a role in the regulation of litter size in pigs.

At current state it is only possible to hypothesize about possible biological mechanisms related to the imprinted (DIO3) QTL. The most plausible explanation is that DIO3 could play a role in the regulation of female fertility and/or on the survival of fertilized oocytes and embryos.

Limited studies have described the effect of imprinted genes on litter size. An imprinted effect on litter size has been observed in mouse for the (predominantly) maternally expressed gene GRB10 [48]. Larger litters, smaller offspring and reduced placenta size was observed in female mice receiving an inactive GRB10 allele from their mothers as compared to inheriting an inactive GRB10 allele from their fathers. For GRB10, the difference in mean mouse embryo weight/offspring at day 17.5 was 6.8% which is in line with the difference in mean TB birth weight/offspring of the two heterozygotic classes for SNP ASGA0037226 in both C1 4.1% and C2 9.6%. Thus, the effect of the two imprinted genes GRB10 and DIO3 is remarkably concordant, suggesting a possible general role for imprinted genes in litter size likely through regulation of placental and/or fetal growth.

The genotypic effects for the imprinted QTL suggest maternal expression (according to the classification of Wolf et al. 2008 [37]). This suggest maternal expression of DIO3 which is opposite to the (partial) paternal gene expression observed for DIO3 in mouse and pig [41]–[46]. Where the paternal expression of DIO3 in mouse and pig was found in fetal/infant stages of development the imprinting effect that we observe is likely to be expressed in the uterine tissue of the mother. This suggest that DIO3 in pigs have different tissue-specific modes of parental expression. Such reciprocal imprinting has also been observed for GRB10 in both human and mouse [13], [14], with reverse imprinting between e.g. embryonic brain and placental tissue.

The similarities in partial and reciprocal imprinting of both GRB10 and DIO3 is notable. Assuming that larger litters place a greater demand for resources on the mother, these similarities may indicate that parental regulation of the imprinting level of these genes are still under natural selection for optimal parental regulation of resources to the offspring(s) as predicted by the parental-offspring conflict hypothesis for genomic imprinting [31].

The higher than expected frequencies of the BA genotype of SNP marker ASGA0037226 in both populations was of interest because this genotype class was also favorable in terms of the traits studied in both populations (sows with a BA genotype had more offspring than sows with a AB genotype (Table 6)). The reason of the relative excess of this genotype class is unknown, but it could be argued that, in addition to the imprinting effect of this marker on reproductive performance, this marker may also have a direct effect on the individual itself on e.g. survival. To check this, the relative frequency of the BA genotype class across parities was calculated for both populations. Since the relative frequency remained constant across parities, it seems unlikely that sows with a BA genotype have a better survival than sows with a AB genotype.

Recent publications reported an effect of the paternally expressed IGF2 gene on sow prolificacy traits [49], [50]. In the present study, the significance of imprinting effects of SNP in IGF2 region did not pass the threshold (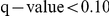): the most significant imprinting effect on TB in region 2_1 had a p-value of 0.016 in population C1 and 0.045 in population C2 and the most significant imprinting effect on LB was 0.011 in population C1 and 0.068 in population C2. The percentage of the phenotypic variances explained by region 2_1 were also much lower than the percentage of variance explained by region 7_1. These results clearly indicate the importance of a possible imprinted gene located in region 7_1 on litter size traits.

## Materials and Methods

### Selection of imprinted regions and SNP markers

In this study, we only considered imprinted genes which have been experimentally confirmed in human, mouse or other mammalian species. These more than 100 imprinted genes are located in 40 regions on the human genome (based on information available at the time the study was designed, i.e. December, 2008). Fifteen of these regions were selected for genotyping (see supplemental file S1). The regions were selected based on the following criteria. 1) An orthologous region should be present in the pig genome (pig reference genome build 7 or 8) or on a pig BAC clone (NCBI High throughput genomic sequence database). 2) Phylogenetic conservation of imprinting; evidence for imprinting found in both human and mouse, and preferably also in pig or in another cetartiodactyl. 3) Strength of imprinting evidence; imprinting reported in more than one publication. 4) Number of imprinted genes in the region; preferably more than one gene is imprinted in the region. 5) By tissue specific imprinted genes; the imprinted gene should preferably be imprinted in a certain stage of reproduction and embryonic/fetal development. 6) Gene function of the imprinted gene; the imprinted gene should play a role in reproduction or in embryonic or fetal development.

The location of the regions in the pig genome, orthologous to the imprinted regions in human plus 0.25 Mb at the 5′ and 3′ flanking sequence, were found by megaBLAST searches [51] against the pig reference genome (build 7 or 8) or pig BAC clones. The megaBLAST searches were done with either pig mRNA/ESTs orthologous to the human genes present in the imprinted region or if no pig orthologous was present with human and/or cow gene sequences. The regions were named according to the chromosome on which they occur and to their order on each chromosome (see Table 3).

A 384-plex Golden gate SNP assay was developed to cover the fifteen selected regions. Twenty to 38 SNPs were allocated to each region. The number of SNPs allocated to the different regions depended on the number of imprinted genes in each region, on the size of the region and on the expected importance of the imprinted genes in the region on reproduction. (see Table 3 for an overview of the regions). The SNPs were selected from the SNP discovery panel which was used to design the Illumina Porcine 60K-chip [52]. A number of criteria were used to select the SNPs. 1) SNPs were as equally as possible dispersed over a region, based on their position in the pig reference genome (version 8) or BAC clone. 2) SNPs with high Illumina design score (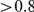) were preferred, as were SNPs with a high minor allele frequency in the SNP discovery panel.

### Population and phenotypes

In the association study, sows from two purebred lines of the Dutch breeding companies Hypor (further denoted as population C1) and Topigs (further denoted as population C2) were genotyped and their data were analyzed with the objective to detect genomic imprinting affecting reproduction traits. These populations were chosen because they had detailed information on fertility traits and because they were sufficiently large to allow for optimization of the study design.

To enable accurate inference of allele origin, which involves inference of haplotypes, a sow was only selected when her father and more than two of her paternal halfsibs were available for genotyping. Available ancestors of a selected sow were also selected for genotyping.

The pedigree of population C1 consisted of 6750 individuals, of which 4033 had phenotypes and in total 689 individuals from this population were genotyped. The pedigree of population C2 consisted of 10096 individuals, of which 3297 had phenotypes and in total 1050 individuals from this population were genotyped. On average, 4 generations of pedigree were available for the genotyped individuals of population C1 and 6 generations for the genotyped individuals of population C2.

The phenotypes considered in this analysis were the total number of piglets born (TB), the number of piglets born alive (LB), the total weight of the piglets born in kilograms (TW) and the total weight of the piglets born alive in kilograms (LW). The weight traits TW and LW were expressed in kilograms and fewer observations were available for these traits than for the count traits TB and LB.

The records of litters until the fourth parity of a sow were used in the analyses. A record of a specific trait was considered as outlier and excluded from the analyses when it deviated more than three standard deviations from the mean of that population. In population C1, 92 records for TB, 136 for LB, 10 for TW, and 8 for LW were considered as outliers. In population C2, 97 records for TB, 97 for LB, 43 for TW, and 35 for LW were considered as outliers. Outliers were removed because one outlier can have a dramatic effect on the p-values, in case outliers occur in genotype classes with only a few observations. On the other hand removing outliers might result in missing interesting findings. Therefore we compared for each company if genotype frequencies in the outliers and the data that was analyzed differed. This was not the case suggesting that outliers were randomly distributed across genotype classes. In addition, records for all four traits of a specific litter were excluded when TB or LB of that litter were 0. In population C1, no records were excluded for this reason. In population C2, the records of 712 litters were excluded for this reason.

### Isolation of DNA and beadexpress genotyping

Samples from the two pig populations were supplied as hair or blood samples by the two breeding companies. DNA was isolated either from hair with the NucleoSpin tissue kits or from blood with the NucleoSpin blood kit, following the instructions of the manufacturers. The DNA concentration was determined with a NanoDrop Spectrophotometer and diluted or concentrated by evaporation to a working concentration of 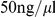 for genotyping. SNPs were genotyped with the Illumina GoldenGate assay and run on an Illumina BeadXpress according to the manufacturer's protocols (http://www.illumina.com). The Illumina's GenomeStudio 2009.1 framework Genotyping Module (v1.0) was used to score genotypes from the raw BeadXpress data. A manually refined genotype clustering file, based on 192 samples, was used for genotype scoring and the 384 SNPs were inspected to detect erroneous SNPs, which were excluded from further analyses. After excluding erroneous and monorphic SNPs, 309 SNPs remained for the association study.

### Genotype correction and haplotype inference

Mendelian inconsistencies in the genotype data were identified using the program Mendelsoft [53], [54] and the critical genotypes suggested by this program were set as missing. The program Mendelsoft identifies the genotypes which most likely are erroneous based on the genotype data of the whole pedigree [53], [54]. From population C1, 1759 of the 245088 genotypes were set to missing and from population C2 716 of the 358974 genotypes were set to missing.

The parental origin of alleles were estimated using the program cvmhaplo [38]. This program estimates the haplotype configuration of the genome segment of interest by optimizing the probability of this configuration given the complete pedigree, i.e. including non-genotyped individuals [38], and based on the assumption that the recombination rate in a segment is proportional to the length. Due to the computational limitations related to the large and complex pedigree, the program was run on overlapping segments of at maximum six consecutive markers. The program was run for each population separately.

### Models

#### Statistical analyses

The univariate statistical analyses of the data were performed for each population and each trait separately. The following mixed effects model was fitted to the data using ASREML [55]:
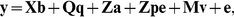
(1) where **y** is a vector of phenotypic observations, **X** is the design matrix of the fixed effects, **b** is an unknown vector of fixed effects, **Q** is the design matrix of the effects of a specific marker which is explained below, **q** is an unknown vector of additive, dominance and imprinting effects of that marker. Matrix **Z** is the design matrix of the random additive genetic effects **a** and of the permanent environmental effects **pe**. A multivariate normal distribution with covariance matrix 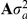 was assumed for the vector of additive genetic effects **a**, were **A** is the additive genetic relationship matrix calculated from the pedigree. A multivariate normal distribution with covariance matrix 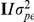 was assumed for the nongenetic permanent environment effects **pe**. Matrix **M** is the design matrix for the maternal effects, i.e. the mothers of the sows in our data. A multivariate normal distribution with covariance matrix 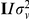 was assumed for the unknown vector of maternal effects **v**. A multivariate normal distribution with covariance matrix 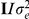 was assumed for the vector of residuals **e**.

The fixed effects included in the model (apart from the marker effects) were a class effect accounting for the breed of the litter (identical to the breed of the service father since all sows within a population were from a single breed) (six levels in population C1 and 13 levels in population C2); a class effect accounting for parity of the sow (four levels in both populations); and a class effect accounting for the combination of farm, year and season (135 levels in population C1 and 333 levels in population C2).

In an initial analysis, the model without the marker effects (the 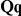 term in Equation 1) was fitted separately to the data of populations C1 and C2 in order to estimate variance components 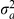, 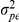, and 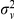. In subsequent analyses, the model including the marker effects was fitted for each marker separately while fixing the variance components to the obtained estimates.

#### Modeling marker effects

Design matrix **Q** in Equation 1 has dimensions equal to n rows, corresponding to the number of observations in the data, and 3 columns, corresponding to the additive, dominance and imprinting effect of a specific marker. Matrix **Q** was calculated as 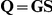, where **G** is a n by 4 matrix denoting the four genotype classes (AA,BA,AB,BB) to which each genotype belonged. In this notation, the first letter of the genotype indicates the allele inherited from the mother and the second letter the allele inherited from the father. Matrix **S** is a 4 by 3 contrast matrix of the additive, dominance and imprinting effect, as used by Hager et al. [36]:
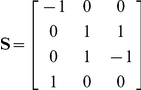



The first column of **S** corresponds to the additive effect, the second column of **S** corresponds to the dominance effect and the third column of **S** corresponds to the imprinting effect. The four rows of **S** correspond to the four genotype classes.

Incremental F-ratios were calculated for the additive, dominance and imprinting effects of each marker, including the marker as the last fixed effect in the model. Following the decomposition of genetic variance by Fisher [56], the dominance effect was included after the additive effect, and the imprinting effect was included after the dominance effect. This order corresponded with the order of the columns of **Q**.

The significances of the marker effects where tested using the F-test statistic and the Kenward and Roger approximation for the denominator degrees of freedom as calculated by ASREML [55] using fixed variance components. To avoid the large number of false positive test results due to the large number of tests performed, the false discovery rates (FDR) were calculated, following the description of Storey and Tibshirani [57] and using the R-package qvalue [58]. We used the term 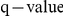 to report the significance of an effect expressed as its FDR.

The q-values were calculated separately for each combination of population, trait, and genetic effect (additive, dominance, and imprinting). The strength of evidence was expressed as the q-value of the test, following the notation of Storey and Tibshirani [57]. Tests with a 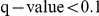 were considered significant.

## Supporting Information

Supplemental File S1 **Infomation of the markers and P-values for each marker.** The list of markers shows the markers included in the analysis, with their position on the reference genome build 9, the region in which they were located and other information. The list of P-values of the markers shows the P-value for the Additive (A), Dominance (D) and Imprinting (I) effect of each marker in each analysis (four traits x two breeding companies).
(XLS)
